# Parents’ Perceptions of the Factors Influencing the Uptake of Remote Pediatric Hearing Aid Support: Development of a Conceptual Framework

**DOI:** 10.2196/47358

**Published:** 2023-06-06

**Authors:** Silva Kuschke, Sheila Moodie, Keshinisuthan Kirubalingam, Robin O'Hagan, Danielle Glista

**Affiliations:** 1 hearX SA (Pty) Ltd Pretoria South Africa; 2 School of Communication Sciences and Disorders Faculty of Health Sciences The University of Western Ontario London, ON Canada; 3 National Centre for Audiology The University of Western Ontario London, ON Canada; 4 Department of Otolaryngology – Head & Neck Surgery The University of Western Ontario London, ON Canada

**Keywords:** audiology, care, child engagement, children, concept mapping, cost, hearing aid, hearing loss, hearing, integration, parents, pediatric audiology, pediatric, remote hearing aid support, support, virtual care

## Abstract

**Background:**

To achieve effective integration of virtual care into family-centered audiology practices, participatory research methods, including parents as vital participants in the delivery of pediatric audiology care, should be considered. A better understanding of the barriers and facilitators influencing the adoption of virtual care for families is warranted.

**Objective:**

This study aimed to develop a conceptual framework of the factors perceived to influence the adoption of remote pediatric hearing aid support among the parents of children with hearing loss.

**Methods:**

A total of 12 parents of children who wear hearing aids, between the ages of 0-17 years, were recruited to participate in group or individual interviews as part of the 6-step participatory-based concept mapping (CM) process. Data collection was specific to parents in a Canadian context. Analyses included multidimensional scaling and hierarchical cluster analysis.

**Results:**

The CM process resulted in 6 main themes, displayed in a cluster map according to their order of importance. These themes include access to timely, consistent care; technology considerations; convenience; child engagement; cost; and partnership considerations. Key underlying statements and subthemes are highlighted per theme.

**Conclusions:**

Findings from this study demonstrate the use of CM in participatory research with parents and as part of a family-centered care model. Future research should aim to investigate the factors that influence the uptake of remote hearing aid support in different contexts, for example, in low- to middle-income countries versus those in high-income countries.

## Introduction

Clinical interactions occurring remotely have been slowly increasing in audiology practice over the past few decades, with a rapid increase observed since the onset of the COVID-19 pandemic to improve access to audiological services [1-3]. Remote delivery of hearing health care services can be accomplished using a virtual care delivery model. Within this study, virtual care describes interactions among families, patients, and audiologists delivering follow-up hearing aid services remotely using any forms of communication or information technologies with the aim of facilitating or maximizing the quality and effectiveness of the care process [4]. According to recent survey findings reported by Eikelboom and colleagues [2], international audiologists exhibit more positive attitudes toward and a greater use pattern around virtual audiology care following COVID-19, pointing to the need to integrate virtual care as part of the new normal. This study aimed to contextualize virtual care within Canadian hearing health care, with a focus on the provision of family-centered care.

For young children, early intervention for hearing loss significantly improves speech and language development trajectories, helping to ensure that children are ready for school entry [5]. Limited access to early hearing detection and intervention services may negatively impact speech, language, and other important early developmental milestones [6]. For families of children with hearing loss, virtual care has been shown to reduce the rate of loss due to early hearing loss detection follow-up appointments without affecting level of satisfaction with service delivery [7]. Family members are often included in virtual care appointments as patient-site facilitators with varying roles [8]. The benefits of family-centered engagement in virtual care are starting to appear in the hearing health care literature. For example, Muñoz et al [9] reported improved parental knowledge, confidence, and abilities to manage their child’s hearing aid following a randomized control trial on the topic of eHealth parent education specific to hearing aid management intervention. The literature also reported positive parent experiences with virtual support interventions, indicating that the sessions they were provided with were effective for supporting their partnership with service providers [10]. Overall, virtual care can benefit families through the provision of flexible and timely access to support, and as part of pediatric hearing aid management care, it has been reported to improve hearing aid use [11].

Central to the development of listening and spoken language is the use of hearing aid technology to support child development. Within virtual environments, barriers still exist when it comes to the effective integration of hearing technology, calling for a better understanding of the implications for parents and families and the range of support required to facilitate virtual care [10]. From the clinician perspective, barriers to effective virtual care delivery may include the quality of the patient-provider relationship, technology limitations at the patient or remote site, digital literacy, and the need for additional training as significant barriers to the delivery of virtual audiology [12,13]. When it comes to technology-related considerations, key clinical factors influencing the use of virtual care include the integration of accessible and easy-to-use technology, a robust internet connection, and the provision of support around the setup and maintenance of equipment [14]. There is a gap in the literature around the key factors perceived to influence family-centered virtual care, including remote hearing aid support.

**Figure 1:**
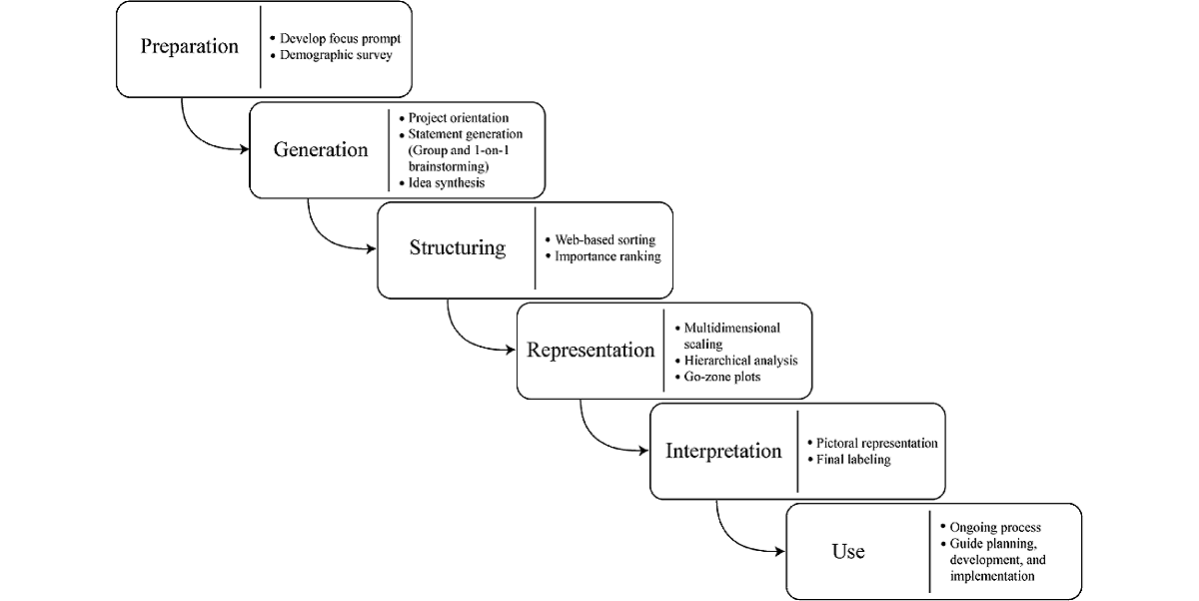
The 6-step concept mapping process as integrated in this study.

Engaging families and community partners in the research process have been identified as a feasible and effective tool for obtaining a broad range of input to identify priorities in intervention approaches [15]. Concept mapping (CM) is emerging as an increasingly useful methodology for implementation science and participatory-based research [16]. This collaborative approach to research can be useful to ensure successful integration of novel intervention procedures by identifying barriers to uptake [17-19]. Within the field of communication sciences and disorders, CM has been used to better understand the barriers and facilitators associated with the uptake of evidence-based services [14,20]. Recently, Glista et al [14] used CM to help identify factors perceived by audiologists to be significant in the adoption of remote hearing aid support services, as a sister project to the study presented within this paper. One of the inherent strengths of CM is that participants are directly involved in the data analysis process, helping to drive the interpretation of findings and discussions [21,22]. The structured process of CM helps to produce results that directly reflect the thoughts and ideas of the participants, with a focus on 1 topic of interest and the integration of participant input to produce an interpretable graphic view of interrelated ideas [21,23,24]. Traditionally, CM involves a 6-step mixed methods process; this process, as well as description of all related tasks per step as implemented in this study, are depicted in Figure 1.

To date, there have been few studies conducted with parents that examine their perspective of virtual audiology services as well as a knowledge gap specific to remote hearing aid support. This study aims to fill this knowledge gap to help guide family-centered virtual hearing aid care, an integral part of early hearing detection and intervention programs, by examining the factors that influence Canadian parents’ use of remote hearing aid support. This work is timely as virtual hearing aid services are continuing to expand globally, and it is incumbent that we understand how to achieve effective delivery in family-centered care models.

## Methods

### Study Design

**Figure 2:**
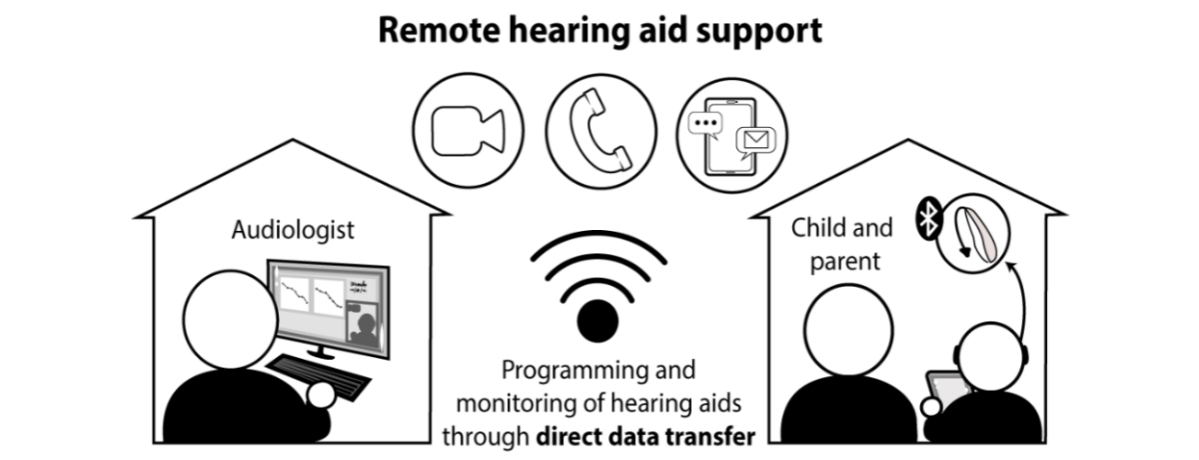
An illustration of remote hearing aid support, connecting an audiologist at a clinic site to a family at home.

In this study, CM methodology was used to develop a conceptual framework of the factors that influence adoption of virtual audiology practices by parents of children with hearing loss and the provision of follow-up hearing aid support. Figure 2 illustrates this application of virtual care and a typical interaction that can result between an audiologist at a clinic-site and a family (eg, child and parent) at their home location.

### Participants

The participant recruitment process was guided by selecting appropriate study participants based on the research goals [22,25]. Convenient purposive sampling was used to recruit parent participants until no new statements were generated during the brainstorming phase. Details added to Methods section: there is no specific number of participants recommended for a CM study [26]. Kane and Trochim [27] suggest that least 5 participants can produce meaningful data. Parents of children aged 0-17 years who have hearing loss, who were fitted with hearing aids, and who reside in the province of Ontario, Canada, were included. Parents were required to have had experience participating in audiology appointments related to their child’s hearing aid fittings; have access to a computer and the internet at home; and be proficient in English. Parents were recruited from a database within Western University’s National Centre for Audiology or through professional networks. Recruiting audiologists were given a letter of information to pass on to potential parent participants. Interested parents were asked to contact either the principal investigator or another member of the research team. Parents were invited to participate in a face-to-face group setting (n=3) or as part of a 1-on-1 telephone interview (n=9) during the timeline of June 2018 to June 2019. Participant characteristics are summarized in Table 1, including information related to the audiology care setting experienced by the family and parent-specific technology use.

**Table 1:** Participant characteristics as reported by the parents.

Participant characteristics			Participants (N=12), n (%)
**Parent age (years)**			
	30-49	9 (75)	
	50-64	3 (25)	
**Parent gender**			
	Male	0 (0)	
	Female	12 (100)	
**Audiology care setting**			
	Private practice	2 (17)	
	Hospital	4 (33)	
	College or University	8 (67)	
	Mobile clinic	1 (8)	
**Technology owned**			
	Smartphone	12 (100)	
	Tablet	10 (83)	
	Desktop	5 (36)	
	Laptop	12 (100)	
**Computer knowledge level **			
	Beginner	1 (8)	
	Average	8 (67)	
	Advanced	3 (25)	
	Expert	0 (0)	
**Ability to use smartphones or tablets **			
	Beginner	1 (8)	
	Average	10 (83)	
	Advanced	1 (8)	
	Expert	0 (0)	
**Ability to download “apps” on a smartphone or tablet**			
	Beginner	2 (17)	
	Average	7 (58)	
	Advanced	3 (25)	
	Expert	0 (0)	
**Current use of virtual care **			
	Yes	2 (17)	
	No	10 (83)	
**Child age (years) **			
	0-3	0 (0)	
	4-7	1 (7)	
	8-11	5 (36)	
	12-17	8 (57)	
**Child identity**			
	Male	8 (67)	
	Female	4 (33)	
**Child attendance in care appointments**			
	Yes	12 (100)	
	No	0 (0)	
**Child-led technologies**			
	Smartphone	8 (57)	
	Tablet	11 (92)	
	Desktop	4 (33)	
	Laptop	10 (83)	

### Ethics Approval

This study was part of a sister project also exploring the factors that influence the uptake of remote hearing aid follow-up support by clinicians [14]. Both studies were approved by the Health Sciences Research Ethics Board of the University of Western Ontario (approval number 109403). All participants provided written consent to participate in either a face-to-face session or a telephone interview, as well as several follow-up web-based tasks using a personal computer.

### Procedures

Participants completed web-based tasks, including sorting and rating, using the Concept Systems Global Max software [28]. The Concept Systems Global Max software uses CM methodology with a web-based interface and is based on group process techniques [29]. To enable web-based tasks, participants were invited through email, in which a weblink to the CM software was provided. A paper record of a unique login and password was provided to each participant.

### Preparation

All participants were asked to complete a short demographic survey that was delivered in person for face-to-face session attendees and over the phone for telephone interviews. Focus prompt development was consistent with that of the sister project, using expert opinion in the development [14].

### Brainstorming and Idea Synthesis

Each face-to-face session began with a brief orientation presentation on the topic of remote support in audiology, sample applications, the use case of interest (follow-up hearing aid support), as well as a summarization of the study methods. Before telephone sessions, the orientation step was delivered using a short, animated 4-minute video created in VideoScribe. The video was accessed by the participants through an emailed weblink. During all sessions, participants were asked to develop as many statements as possible to complete the CM focus prompt: “One thing that may influence my use of teleaudiology for remote follow-up hearing aid support is…” A synthesis step was used by the investigators to combine the final statements into 1 large data set and eliminate redundancies. The decisions made during the synthesis step were manually recorded by the investigators, resulting in a list of key statement synthesis decisions. The face-to-face sessions and phone interviews were also audio-recorded.

The researchers compiled all the statement sets into 1 large set that was then edited and synthesized by the research team to eliminate redundancies and refine statements to ensure clarity and comprehension. Statements that were unrelated to the prompt were removed; statements were also merged or split to ensure that each statement had 1 clear meaning [25]. The statement synthesis steps were recorded to create an audit trail of the consolidated set of 125 statements. The final (synthesized) set included 107 unique parent statements related to the focus prompt.

### Structuring

Participants were contacted twice through email to complete web-based follow-up tasks. Overall, 8 participants completed the web-based sorting task and 9 completed the importance ranking task. A web link to the Global Max software was sent in the first follow-up email. Instructions were given within the software to facilitate the sorting of the final set of statements into piles and the ranking of the statements based on perceived importance. The importance-ranking instructions provided to the participants were as follows: “Please rate each statement on a scale of 1 to 5 where; 1=relatively unimportant; 2=somewhat important; 3=moderately important; 4=very important; 5=extremely important. To complete each rating, type a number next to each statement.”

Following analyses, the research team conducted a final review of the statements, clusters (which are resultant categories of similar ideas generated in the brainstorming phase), and corresponding names of each cluster, with the end goal of achieving group consensus on acceptable labels for each cluster and number of clusters. This final step was mainly driven by the research team due to the outbreak of the global COVID-19 pandemic, which limited contact with the participants.

## Results

### Representation

**Figure 3:**
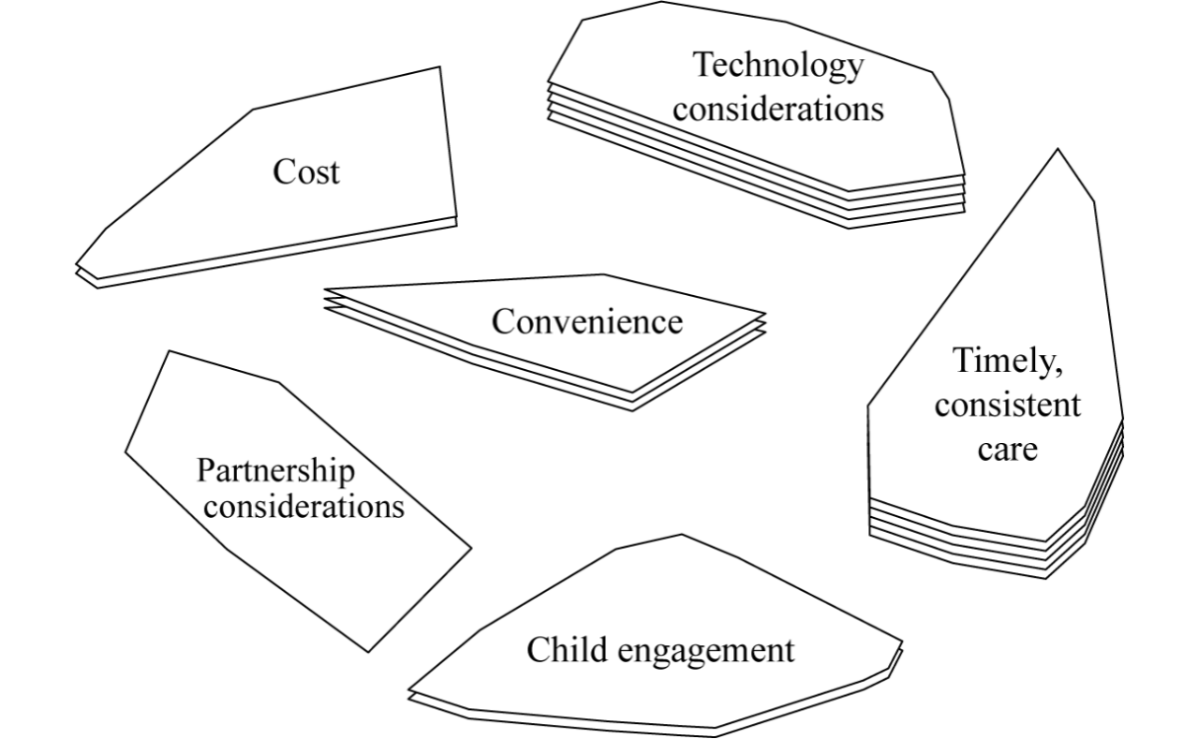
Six-cluster map of the 107 statement point map, of factors influencing the uptake of remote hearing aid support by parents labeled by importance rating.

The representation of results was enabled using the Global Max software and used 2 types of analyses: multidimensional scaling and hierarchical cluster analysis. Details regarding the production of the final concept map are described in the representation section. Multidimensional scaling was used to locate each parent statement in a 2D space to display on a point map, following a 2-step process from Kane and Trochim [27]: (1) A similarity matrix, using a similarity cutoff of 3 to filter out false relationships between statements, was generated by pairing the 107 statements with one another and assigning a numerical value indicating the number of parents who put that pair of statements in the same pile; and (2) A 2D solution was used to produce x- and y-coordinates for each statement, following a bivariate distribution. These steps resulted in the generation of a point map that was used to yield a 6-cluster configuration; this cluster map is illustrated in Figure 3. Each point on the point map represents 1 statement. Statements that were sorted together more often by the participants appear as points closer together; statements that were less often sorted together appear further apart [30]. A stress value is a statistic routinely generated and reported in multidimensional scaling analyses, indicating how well the statement configuration matches the data [21]. The final stress value of 0.34 falls within the normal and acceptable range for CM research, indicating that the map appropriately represents the sorting data [27]. Also known as a concept map, the resulting cluster map helps to depict a group-level conceptualization of parent-generated ideas around the factors influencing the uptake of remote hearing aid support. The final stress value of 0.34 falls within the normal and acceptable range for CM research, indicating that the map appropriately represents the sorting data [27]. 

Hierarchical cluster analyses, using input from multidimensional scaling, mathematically grouped each statement into adjustable cluster configurations, based on how parents rated and sorted the data. Each cluster represents a unique theme on the resulting map (Figure 3). The selection of the final number of clusters included in the CM required both software and researcher input to yield an optimal solution. The recommendation is for researchers to examine a range of possible clusters suggested by the software program, consider the statements included within the cluster, and use this information to formulate a cluster configuration [27]. Possible cluster configurations were considered and discussed by the research team (DG, SM, and RO) and a final configuration of 6 clusters was selected. 

### Interpretation

Final cluster labels reflect the general theme for each cluster of statements. The layers per cluster indicate the level of importance rating provided by the parents for their statements per cluster. A greater number of layers represents greater importance for the group of statements included within the cluster, as judged by individual parents. For example, access to timely, consistent care was rated by the parents as more important when compared to partnership considerations.

A list of the main themes, subthemes, and example statements were generated using single cluster go-zone plots, allowing for visualization of the relationship between participant ratings with respect to the concept map (Table 2). The example statements presented by theme included those that received high overall average ratings of importance. Table 3 provides a count of the total number of statements that appear in each cluster, along with the mean importance values for each cluster. Mean cluster values are presented in order of importance from most to least important and correspond to responses collected using the 5-point scale.

**Table 2:** Concept mapping clusters, subthemes, and example statements created using the prompt: “One factor that will influence my uptake of remote hearing aid follow-up support is...”

Cluster	Overall subthemes	Example statements
Access to timely, consistent care	Participation of relevant stakeholders and consistent access to remote audiological services	“Ability to troubleshoot remotely in listening environments important to my child.” “The ability to access remote fitting support in times of need (eg, during extracurricular activities, hearing aid emergencies).”
Technology considerations	Access to stable and consistent technology (equipment and internet access); training and support available	“Access to a phone/tablet that is current enough to be used for remote fitting appointments.” “The learning curve associated with remote hearing aid appointments.”
Convenience	Traveling distance; time taken off from work and school	“If I don’t have to miss work for an appointment.” “If my child doesn’t have to miss school for an appointment.” “If it eliminates the need to arrange for child-care at the time of an appointment.”
Child engagement	Child’s, parent’s, and audiologist’s confidence and involvement in appointments	“If it builds/maintains a positive relationship between my child and his/her Audiologist.” “If it addresses my child’s need for frequent follow-up appointments following a new hearing aid fitting.”
Cost (financial and otherwise)	Costs associated with the appointment in terms of equipment and traveling	“If the cost of a remote appointment is the same or less than a face-to-face appointment.” “The need to negotiate with family members to use a phone/tablet for a remote appointment.”
Partnership considerations	Privacy considerations; participation of family members and other health care professionals in the appointment	“The ability to have other healthcare professionals participate in an appointment.” “The quality of the services being delivered remotely, in comparison to face-to-face.”

**Table 3:** Clusters and corresponding total statement numbers arranged by importance level, along with the overall mean importance values.

Cluster	Statements, n	Importance values, mean (SD; range)
Access to timely, consistent care	22	4 (0.51; 3.1-4.9)
Technology considerations	24	3.93 (0.57; 2.2-4.7)
Convenience	13	3.71 (0.68; 2.3-4.6)
Child engagement	23	3.65 (0.69; 2.6-4.8)
Cost (financial and otherwise)	11	3.55 (0.67; 1.8-4.2)
Partnership considerations	14	3.41 (0.96; 1.3-4.7)

## Discussion

### Overview

This study demonstrates how CM was used to develop a conceptual framework in collaboration with parents of children wearing hearing aids to explore factors that may influence the adoption of remote follow-up hearing aid support services delivered through teleaudiology. A total of 12 parents of children who have hearing loss and reside in Ontario, Canada, were recruited to participate in this study. Overall, 6 themes were developed to form the final conceptual framework of the perceived factors that influence the uptake of remote hearing aid support by parents of children with hearing loss. These 6 themes, in order of overall level of importance, were (1) access to timely, consistent care; (2) technology considerations; (3) convenience; (4) child engagement; (5) cost; and (6) partnership considerations.

Access to timely, consistent audiological care was rated by the parents as the most important factor in delivering remote hearing aid support to their children. Subthemes in this cluster highlighted that parents value the ability to access hearing aid support remotely in times of hearing aid emergencies or during extracurricular activities where listening demands are different. Virtual audiological consultations with parents and families of children who have hearing loss have shown benefits in terms of flexibility and timely access to support [10,11]. Furthermore, increased access to audiological care can result in direct, collaborative problem-solving between the parents and the audiologist, which could lead to increased hearing aid use and better hearing outcomes.

Technology considerations were ranked as the second most important factor in the uptake of pediatric remote hearing support. Technological barriers (such as consistent internet connection and availability of training and support) have been cited in the literature as one of the main reasons why remote hearing aid support is not readily adopted worldwide [31]. The top-rated overall statements by parents regarding technological considerations and infrastructure pertained to accessibility of phones or tablets that are up-to-date to support remote hearing aid fitting appointments and the learning curve associated with remote hearing aid appointments. Confidence is a key aspect when considering engagement in remote hearing aid support, and additional support can guide patients in their ability to manage remote technology [32]. Additionally, the technical knowledge of audiologists who engage in remote hearing aid support has emerged as an important factor in the uptake of remote support by pediatric audiologists [14]. This theme highlights the need for developing clinical guidelines to support both parents of children who have hearing loss and pediatric audiologists in the clinical implementation of remote hearing aid support services and clinical resources to assess and guide family-centered training around virtual care.

Patient- and family-centered factors pertaining to service accessibility were also deemed important by parents. These include convenience (traveling distance, time taken off from work and school, and the need to arrange for childcare), cost (related to traveling and equipment needed for remote support), and child engagement (addressing the ’child’s need for frequent follow-up appointments following a new hearing aid fitting). Teleaudiology has been reported as a cost-effective manner of service provision [33]. In addition, increased convenience and decreased cost could lead to fewer missed appointments [14]. Access to more frequent remote follow-up appointments for the pediatric population could, in turn, minimize the detrimental effects of hearing loss on speech and language development in children [34].

The final cluster pertained to participation and the collaboration between the professional, the parents, and their family members. Audiologists play a vital role in partnering with parents and families to provide the support needed for the effective day-to-day management of their child’s hearing aids [11]. Building a trusting parent-professional partnership helps to implement and sustain consistent daily hearing aid use, which can support developmental outcomes for children.

### Usage

The use step in a CM framework is an ongoing process related to the study objectives and involves working with the stakeholder team to determine the best ways to use the maps and reports produced as part of the CM procedures [30]. A total of 6 main themes and related subthemes emerged from this study, which focused on parent-perceived factors influencing the uptake of remote hearing aid support for their children with hearing loss. Recommendations arising from the identified themes will ultimately be used to help guide the planning, development, and implementation of teleaudiology services, such as remote hearing aid support, into pediatric clinical practice. Planning and implementation of pediatric remote audiological care should be tailored according to a parent and family focus. Results of this CM study reinforce the need for standardized pediatric telehealth protocols and procedures to facilitate remote audiological follow-up care for children [35]. In addition, the CM framework in this study will be used to support future research, including development of best-practice guidelines and training documents to assist in the uptake of remote hearing aid support services for parents and families of children with hearing loss.

### Limitations and Future Research

Study limitations include the use of nonrandom sampling, which results in a sampling bias; a small sample size, which limits generalizability; the labor-intensive process of creating a CM framework; and the personal attributes of the participants, which likely shaped the resulting themes in the framework of this study [36]. Furthermore, no parents of very young children (0-3 years of age) and only 1 parent with a child in the 4-7 years of age category participated in this study, which limits understanding of parent perspectives across the age spectrum. For example, parents typically need educational and management support when hearing aids are first fitted, but this theme was not identified by the participants in this study. A possible reason for this could be that the parents in this study had more experience and were focused on how virtual care could currently help them. Another limitation for this study was that the final member–checking step (usually done by the participants in a CM study) was not completed due to the outbreak of the global COVID-19 pandemic; this was due to an inability to recruit parents to participate in additional steps during this time. Therefore, the final resultant clusters in the CM framework were not reviewed by the parent participants to obtain feedback.

Results of this study demonstrate the benefit of CM methodology in facilitating parent engagement in research. Through the inclusion of parent participants, greater value is added when considering family-centeredness in the context of virtual audiology care. Future research could use a similar approach to investigate factors that influence parental uptake of remote hearing aid support in different contexts and across different languages, for example, in low- to middle-income countries versus high-income countries, as well as in populations for whom English is not the predominant language.

## Abbreviations

CM: concept mapping
